# Ultrahigh permeance of a chemical cross-linked graphene oxide nanofiltration membrane enhanced by cation–π interaction†

**DOI:** 10.1039/c9ra07109a

**Published:** 2019-12-06

**Authors:** Ruobing Yi, Rujie Yang, Risheng Yu, Jian Lan, Junlang Chen, Zhikun Wang, Liang Chen, Minghong Wu

**Affiliations:** Shanghai Applied Radiation Institute, Shanghai University Shanghai 200444 P. R. China mhwu@shu.edu.cn; Department of Optical Engineering, Zhejiang A&F University Lin'an Zhejiang 311300 P. R. China liang_chen05@126.com; College of Pharmaceutical Chemistry and Materials Engineering, Taizhou University Taizhou Zhejiang 317000 China

## Abstract

Cross-linking with large flexible molecules is a common method to improve the stability and control the interlayer spacing of graphene oxide (GO) membranes, but it still suffers from the limitation of low water flux. Herein, a novel high flux GO membrane was fabricated using a pressure-assisted filtration method, which involved a synergistic chemical cross-linking of divalent magnesium ions and 1,6-hexanediamine (HDA) on a polyethersulfone (PES) support. The membrane cross-linked with magnesium ions and HDA (GO_HDA–Mg^2+^_) exhibited a high water flux up to 144 L m^−2^ h^−1^ bar^−1^, about 7 times more than that of cross-linked GO membranes without adding magnesium ions (GO_HDA_), while keeping excellent rejection performance. The GO_HDA–Mg^2+^_ membrane also showed an outstanding stability in water for a long time. The effects of magnesium ions on the GO_HDA–Mg^2+^_ membrane were analyzed using several characterization methods, including Fourier transform infrared spectroscopy (FT-IR), scanning electron microscopy (SEM), X-ray photoelectron spectroscopy (XPS) and X-ray diffraction (XRD). The results indicated that magnesium ions not only promoted reasonable cross-linking, but also improved the stacking of GO sheets to give lower mass transfer resistance channels for water transport in the membranes, resulting in the ultrahigh permeance of the GO membranes.

## Abstract 2

Chemical cross-linking together with magnesium ions, potentially promoting reasonable cross-linking and improving the water channels of membrane in terms of flatness and surface with low mass transfer resistance.

## Graphical abstract

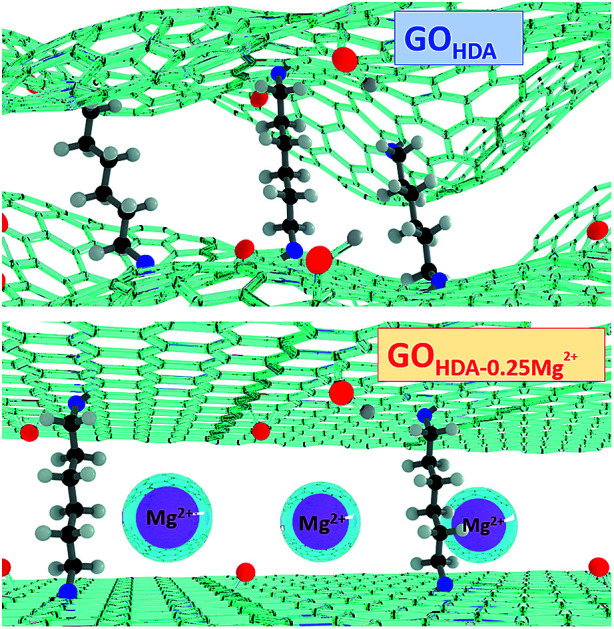

## Introduction

Graphene oxide (GO) is a two-dimensional network with a thickness of one atom, which has great potential in the field of water treatment because of its excellent hydrophilicity,^1^ remarkable stacking property^2^ and other unique properties.^3^ In GO membranes, a large number of oxygen-containing functional groups, such as hydroxyl, epoxy, carboxyl and carbonyl, are randomly distributed outside the pristine graphitic sp^2^ region.^4^ These oxygen functional groups act as water nanochannel spacers to introduce water molecules into the sp^2^ region which can allow water molecules to flow without resistance.^5^ Therefore, GO-based membranes are considered as the next generation of nanofiltration membranes.

In the practical application of GO nanofiltration membranes, the interlayer spacing between the neighboring GO nanosheets, water flux, efficient rejection and stable performance of the membrane are all crucial in water purification.^6,7^ However, the GO membrane is prone to swelling in water due to the large number of hydrophilic oxygen functional groups, which is not conducive to the stable performance of GO membrane in practical application.^8^ There have been many effective efforts to stabilize the interlayer spacing and prevent the swelling tendency of GO membranes. For example, by cross-linking with organic large molecules and ions,^6,9–11^ as well as by reducing the GO membrane to decrease the interlayer spacing, the stability is significant improved.^7,12^ Despite the great progress, these membranes still suffer from limitation of low water flux (<27 L m^−2^ h^−1^ bar^−1^).^2,13,14^ The decreasing of the interlayer spacing and excessive cross-linking between sheets, not only decrease the water channel,^7,10,11^ but also increase the mass transfer resistance,^9^ leading to the low permeance. Therefore, GO membranes is still under the expectation, which requires further increase of water flux without sacrificing stability and rejection.^2^ These challenges hinder the potential applications of GO membranes in water purification.

In our previous work, accurate cationic control of the interlayer spacing of GO membranes with Ångström precision using ions, has been achieved.^11^ The existence of cations adsorbed on GO surface, can greatly improve the flatness of the GO nanosheets, which is conducive to the stacking of GO sheets to form reasonable water nanochannels.^11,15^ Therefore, chemical cross-linking together with ions, potentially promoting reasonable cross-linking and improving the water channels of membrane in terms of flatness and surface with low mass transfer resistance.

For Mg^2+^, it is the most divalent cation and abundant in seawater, which has the same strong cation–π interaction with the graphene sheets as the high multivalence metal ions (Fe^2+^, Co^2+^, Cu^2+^, Cd^2+^, Cr^2+^ and Pb^2+^) have.^16^ Interestingly, GO membranes controlled by Mg^2+^ ions have the largest interlayer spacing compared with other metal ions in seawater.^11^ The large interlayer spacing in GO membrane is the prerequisite for ultrahigh water permeation. However, due to strong cation–π interaction between Mg^2+^ and GO flakes, Mg^2+^ ions can be adsorbed on the GO surface during the cross-linking process, which can prevent excessive chemical cross-linking and improve the water channels of membrane in terms of flatness and low mass transfer resistance surface during the cross-linking reaction.

In this study, the GO membrane with high water permeance for dyes rejection was prepared by pressure-assisted filtration method, which was a synergistic chemical cross-linking of divalent magnesium (Mg^2+^) ions and hexamethylenediamine (HDA) (GO_HDA–Mg^2+^_) on a polyethersulfone (PES) support. We also prepared the cross-linking GO membrane only by HDA for comparison (GO_HDA_).

## Experimental

### Preparation graphene oxide (GO) suspension

Graphene oxide (GO) was prepared from commercial graphite powder by a modified Hummers' method as previously reported.^11,17^ Graphite powders were firstly pre-oxidized by concentrated H_2_SO_4_, K_2_S_2_O_8_, and P_2_O_5_ solution with continuous stirring for 4.5 hours. Secondly the mixture suspension was washed by DI water and vacuum drying for a night. Then, oxide graphite was further oxidized in concentrated H_2_SO_4_ and KMnO_4_, diluted with a lot of DI water. 30% H_2_O_2_ is further trickled to remove excess KMnO_4._ The product was separated by centrifugation and washed with 1 : 10 HCl aqueous solution and DI water. Finally, the GO suspension was prepared after half an hour of ultrasound.

### Preparation cross-linking GO membrane

MgCl_2_ were added into 150 mg L^−1^ GO and stirred evenly. The concentration of Mg^2+^ in the mixed suspension was 0.25 M, 0.125 M, 0.05 M, 0.025 M and 0 M, respectively. Next, 1,6-hexanediamine was dissolved in the above mixed suspension to prepared 0.075 M aqueous solution. The mixtures were left to rest overnight at room temperature, 3 mL solution was dissolved to 40 mL, respectively. Then, they were filtered through a polyethersulfone (PES) membrane under a pressure of 1 bar. Finally, the membranes were washed with 1 : 10 HCl aqueous solution and DI water. The membranes prepared at a series of concentrations of Mg^2+^ were named as GO_HDA–0.25Mg^2+^_, GO_HDA–0.125Mg^2+^_, GO_HDA–0.05Mg^2+^_, GO_HDA–0.025Mg^2+^_ and GO_HDA_, respectively. GO_HDA–0.25Mg^2+^_, GO_HDA–0.125Mg^2+^_, GO_HDA–0.05Mg^2+^_ and GO_HDA–0.025Mg^2+^_ are collectively referred to as GO_HDA–Mg^2+^._

### Filtration experiment

The permeation and rejection performance of the cross-linking membranes were tested by using a vacuum filter system with an effective membrane area of 11.34 cm^2^. The rejection tests were performed with 10 mg mL^−1^ rhodamine B (RB), pararosaniline (PR), and methyl blue (MB) solutions, respectively. The molecular weights of the three dyes are 479.01, 323.82 and 319.86 g mol^−1^, respectively. When filtration went steady, the water flux (*J*_w_) and Rejection (*R*) was measured at 1 bar by using the following eqn (1) and (2):1
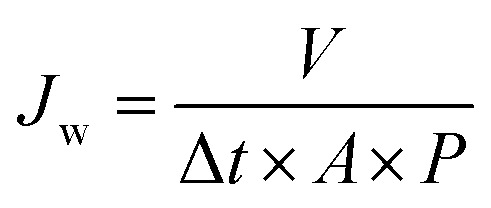
2
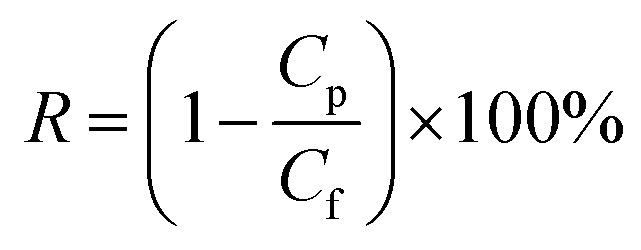
where *J*_w_ is the water flux (L m^−2^ h^−1^ bar^−1^), *V* is the volume of the permeation water (L), *A* is the effective membrane area (m^2^). Δ*t* is the permeation time (h) and the *P* is the filtration pressure (bar). *C*_p_ and *C*_f_ are the concentration of permeation and feed dye solution which were measured by ultraviolet spectrophotometry, respectively.

## Results and discussion

### The chemical properties of GO and cross-linking GO membrane

**Fig. 1:**
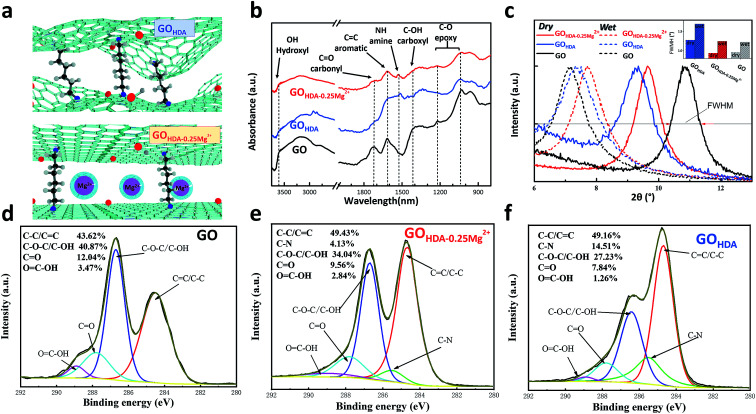
(a) A schematic of GO_HDA–0.25Mg^2+^_ and GO_HDA_; (b) FTIR spectra of GO, GO_HDA–0.25Mg^2+^_ and GO_HDA_; (c) XRD spectra of GO, GO_HDA–0.25Mg^2+^_ and GO_HDA_; X-ray photoelectron spectrometer spectra of (d) GO, (e) GO_HDA–0.25Mg^2+^_ and (f) GO_HDA_.

The FT-IR spectra of the GO, GO_HDA–0.25Mg^2+^_ and GO_HDA_ are shown in Fig. 1b. The chemical structure of GO was clearly changed by HDA. The FTIR spectrum of the pristine GO suggested the presence typical vibrations, such as, the hydroxyl C–OH (stretching at 3594 cm^−1^), carbonyl C=O (stretching at 1730 cm^−1^), carboxyl –OH (bending at 1418 cm^−1^), aromatic (stretching vibrations at 1622 cm^−1^) and epoxy C–O (stretching at 1020–1227 cm^−1^).^18–21^ For GO_HDA–0.25Mg^2+^_ and GO_HDA,_ the peaks of hydroxyl, epoxy and carboxyl decreased dramatically after cross-linking with HDA, and a new absorption peak was observed at 1550 cm^−1^ which represent the bending vibration of N–H.^9,10,22^ The reduction of hydroxyl, epoxy and carboxyl groups and the new generation of amine confirmed that HDA likely reacts with these oxygen-containing groups to form C–N covalent bonds, during the condensation reaction of HDA with hydroxyl^23^ and carboxyl^24^ and the nucleophilic addition reaction of amine with epoxy.^24^ In addition, compared with the GO_HDA_, the GO_HDA–0.25Mg^2+^_ has more oxygen-containing groups and less amine groups, indicating a weaker reduction and lower degree of cross-linking due to Mg^2+^ added in the reaction process.

To further reveal the chemical properties of GO membranes, C 1s of XPS spectra were used to analyze the elemental compositions of the chemical bonds. As shown in Fig. 1d–f, the deconvoluted C 1s spectra were divided into four peaks at binding energies of 284.6, 286.7, 287.8, 288.9 eV, which corresponded with C=C/C–C, C–O/C–*O*–C, C=O and O=C–O, respectively.^7,25,26^ The contents of C–*O*–C/C–O were 40.87%, 34.04% and 27.23% for GO, GO_HDA_ and GO_HDA–0.25Mg^2+^_ respectively, which significantly decreased by cross-linking. And the intensities of O=C–O were also decreased dramatically. Importantly, a new peak appears at 285.5 eV representing the C–N bond,^9,10^ which are 4.13% and 14.51% for GO_HDA–0.25Mg^2+^_ and GO_HDA_, respectively. The results demonstrated that the GO_HDA–0.25Mg^2+^_ has an effective cross-linking similar to GO_HDA_, while a weaker reduction and lower degree of cross-linking, which is consistent with our FT-IR spectra results. It indicates that under the interaction of Mg^2+^, it is beneficial to reasonable cross-linking between GO and HDA. It not only ensures the stability of the membrane, but also facilitates the formation of channels with low mass transfer resistance.

In addition, we used XPS to detect the atomic concentrations. Fig. S2b† shows the survey XPS scans of the prepared cross-linked membranes. We can see that there are no observable Mg^2+^ ion signals. During the filtration, the filtrates were collected when the filtration process went steady (after about 20 min), which can help to rule out the adsorption effect by the membrane. Thus, the high rejection for dyes remained constant with increasing membrane thickness, is mainly due to stable size exclusion effect based on stable chemical cross-linking with HDA and the water channels of membrane improved by Mg^2+^ during the cross-linking reaction.

### Effects of Mg^2+^ on the interlayer spacing of cross-linked GO

As mentioned above, the water channel of membrane is an important parameter for permeation. These membranes were further analyzed by XRD. There were clear shifts of the interlayer spacing (indicated by the Bragg peaks of XRD) relative to the GO membrane that had been immersed in pure water, as shown in Fig. 1c. Immersion in pure water resulted in a GO membrane spacing from 8.5 Å to 12.8 Å, consistent with early reports.^11^ In contrast, the shifts of interlayer spacing of GO_HDA_ and GO_HDA–0.25Mg^2+^_ between dry and wet state were smaller. The interlayer spacing of GO_HDA_ were 9.3 Å and 11.9 Å in dry and wet state, respectively, due to the limitation of newly formed C–N bonds between the GO sheets.^9,10^ Similarly, those of GO_HDA–0.25Mg^2+^_ in dry and wet state were 9.1 Å and 11.4 Å. However, as shown in the inset of Fig. 1c, the full width at half maxim (FWHM) of GO_HDA–0.25Mg^2+^_ is obviously narrower than that of GO_HDA_, indicating that GO_HDA–0.25Mg^2+^_ has better uniformity of the water channel, than that of the GO_HDA_ membrane. Thus, the channel can be shown in the schematic of Fig. 1a. The rippling and wrinkled structure was aligned flattened by Mg^2+^, which is conducive to the stacking of GO sheets to form a surface with low mass transfer resistance for water transport.^11,15^ Therefore, it can be predicted that the GO_HDA–0.25Mg^2+^_ membrane has a relatively high permeance while maintaining high rejection.

### Morphology of the GO and cross-linking GO

**Fig. 2:**
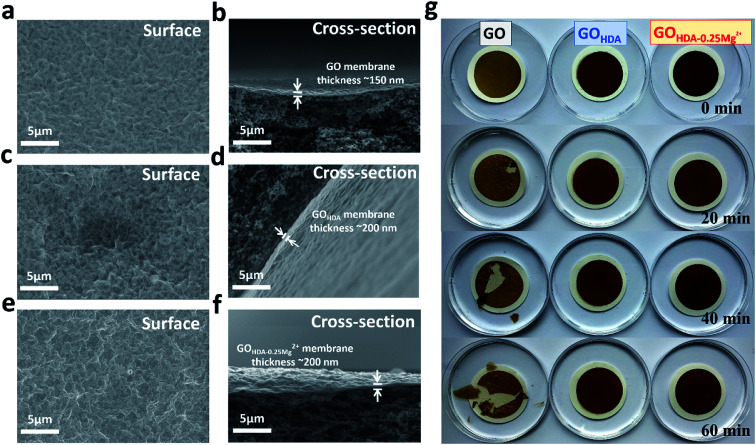
(a) Surface morphology and (b) cross-section morphology of GO_HDA–0.25Mg^2+^_. (c) Surface morphology and (d) cross-section morphology of GO. (e) Surface morphology and (f) cross-section morphology of GO_HDA_. (g) Stability of GO, GO_HDA_ and GO_HDA–0.25Mg^2+^_ membranes in water. These pictures were taken after the water had been stirred with a glass stirring rod.

The Atomic Force Microscope (AFM) image of GO flakes was observed, as shown in Fig. S2a.† The thickness of GO monolayer is about 0.96 nm. Fig. 2a–f show the SEM images of surface and cross-section morphology of the GO, GO_HDA_ and GO_HDA–0.25Mg^2+^_ membranes. The thickness of the cross-linked membrane was about 200 nm, while the thickness of GO membrane was 150 nm. These SEM images showed that the resulting thin layered membrane was continuous and free of macro pores or defects, which is critical for a highly efficient separation process.^11^ As shown in Fig. 2f, GO_HDA–0.25Mg^2+^_ membrane is obviously a multi-layers structure like GO membrane. As shown in Fig. 2g, we also evaluated the stability of the membrane immersed in water. The GO membrane disintegrated after 20 min without mechanical stirring, and then seriously dispersed after 60 min. But GO_HDA–0.25Mg^2+^_ and GO_HDA_ still remain stable, even when the water was stirred by glass rod, indicating that GO_HDA–0.25Mg^2+^_ membrane can overcome the swelling problem in water like GO_HDA_.

### Permeance of cross-linking GO

**Fig. 3:**
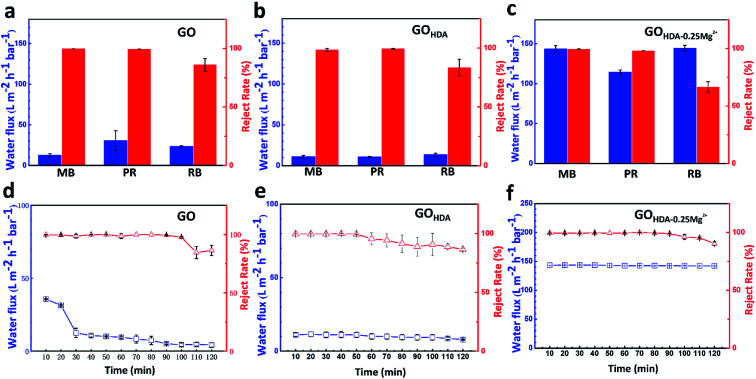
Permeance and rejection of three organic dyes (10 mg L^−1^) of (a) GO_HDA–0.25Mg^2+^_, (b) GO and (c) GO_HDA_ membranes at a transmembrane pressure of 1.0 bar. Flux of the (d) GO_HDA–0.25Mg^2+^_, (e) GO and (f) GO_HDA_ membranes during the filtration of water for 120 min.

We performed dyes permeation tests to verify the water flux and the reject rate of GO, GO_HDA_ and GO_HDA–0.25Mg^2+^_ membranes. As shown in Fig. 3a–c, HDA resulted in a decrease in water flux for rejecting methylene blue, pararosaniline and rhodamine B from 12.5 L m^−2^ h^−1^ bar^−1^, 30.6 L m^−2^ h^−1^ bar^−1^ and 23.5 L m^−2^ h^−1^ bar^−1^ for GO membrane to 11.2 L m^−2^ h^−1^ bar^−1^, 10.8 L m^−2^ h^−1^ bar^−1^ and 13.4 L m^−2^ h^−1^ bar^−1^ for the GO_HDA_ membrane, respectively. But it didn't make much difference to the reject rate. The performance of GO_HDA_ is consistent with early reports.^9,10^ In addition, we also observed the performance of the GO membranes controlled only by Mg^2+^ (GO_Mg^2+^_), which prepared with the same experimental process as did the cross-linking experiments, as shown in Fig. S3.† The water flux of GO_Mg^2+^_ for rejecting methylene blue was 39 L m^−2^ h^−1^ bar^−1^, which is only slightly higher than that of GO. In contrast, GO_HDA–0.25Mg^2+^_ membranes have ultrahigh water flux, which were 143.2, 114.4 and 144.2 L m^−2^ h^−1^ bar^−1^ for the three dyes, respectively, while still rejected dyes as those of GO_HDA_. Interestingly, the water fluxes are nearly 10 times higher than those of GO_HDA_ membranes without sacrificing dyes rejection, as shown in Fig. 3d. It further demonstrated that GO_HDA–0.25Mg^2+^_ membrane has much better uniformity and lower mass transfer resistance than that of GO_HDA_. We also listed the separation performance of GO-based membranes previously reported for organic dyes. As shown in Table 1, GO_HDA–0.25Mg^2+^_ showed great advantage on the water flux.

**Table 1:** Permeation of graphene-based membrane for dyes rejection^a^

Membrane	Dyes	Reject rate (%)	Permeance(L m^−2^ h^−1^ bar^−1^)	Reference
CCG	Methylene blue	>99	3.26–21.81	12
GO + PECs	Methylene blue	99.3 ± 0.1	0.87 ± 0.02	27
TMC + GO	Methylene blue	46–66	8–27.6	14
rGO/MCNT	Rhodamine B	100	52.7	28
NSC-GO	Rhodamine B	87 ± 3	279	29
GO	Pararosaniline	86.48–98.88	11.13–20.23	30
	Methylene blue	90.15–98.97		
GO_HDA–0.25Mg^2+^_	Methylene blue	99.42	143.2	This work
	Pararosaniline	97.90	114.4	
	Rhodamine B	66.54	144.2	

a CCG: chemically converted graphene; PECs: polyelectrolyte complexes; TMC: 1,3,5-benzenetricarbonyl trichloride; MCNT: multi-walled carbon nanotube; NSC-GO: ultrafiltration nanostrand-channelled GO.

The stability of water flux and dyes rejection of GO_HDA–0.25Mg^2+^_ and GO_HDA_ membranes was analyzed. The fluxes were measured for 2 h and recorded every 10 min after adding DI water into the feed side. As shown in Fig. 3d–f, the fluxes of the GO_HDA–0.25Mg^2+^_ and GO_HDA_ were about 114.4 L m^−2^ h^−1^ bar^−1^ and 11.2 L m^−2^ h^−1^ bar^−1^, respectively, which were very stable during the whole filtration process compared with the flux varies from 35.7 L m^−2^ h^−1^ bar^−1^ to 4.3 L m^−2^ h^−1^ bar^−1^ of the GO membrane. It demonstrated that GO_HDA–0.25Mg^2+^_ and GO_HDA_ all have the outstanding stability in filtration process, which is attributed to the C–N bond formed between GO and HDA.

The performance of the GO membranes, which were synergistically cross-linked by K^+^ (GO_HDA–K^+^_) or Fe^3+^ (GO_HDA–Fe^3+^_) were also observed, as shown in Fig. S4.† The membrane cross-linked with K^+^ and Fe^3+^ ions and HDA (GO_HDA–K^+^_ and GO_HDA–Fe^3+^_) exhibited water flux of 40.1 L m^−2^ h^−1^ bar^−1^ and 140.6 L m^−2^ h^−1^ bar^−1^, respectively, while keeping >99% rejection for methylene blue, indicating high multivalence metal ions are beneficial to permeability. Compared with these tested metal cations, the Mg^2+^ ions still have the greatest advantage in terms of water flux.

### Effect of thickness and concentration of Mg^2+^ on permeance of cross-linking GO membrane

**Fig. 4:**
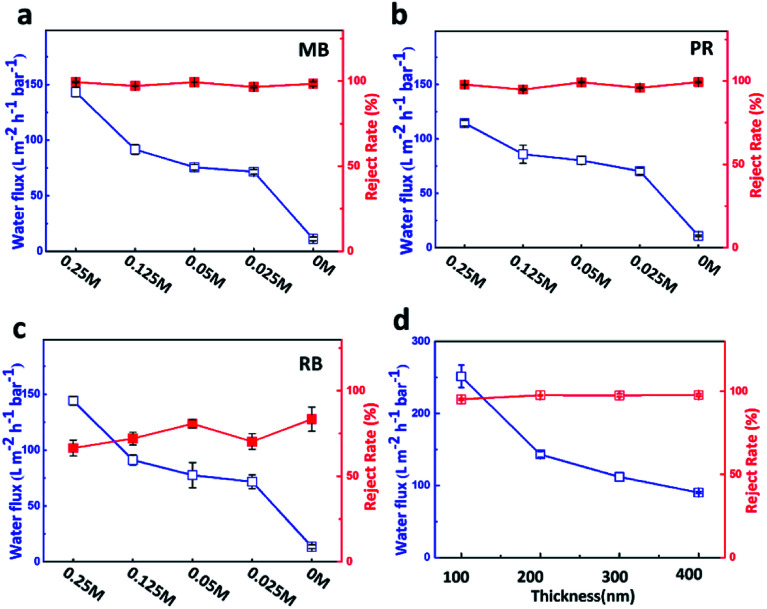
Permeance and reject rate of (a) methylene blue, (b) pararosaniline and (c) rhodamine B (10 mg L^−1^) by GO_HDA–Mg^2+^_ membrane formed with different Mg^2+^ concentrations solutions. (d) Permeance and reject rate of methylene blue of GO_HDA–0.25Mg^2+^_ membranes with different thickness.

It is clear that Mg^2+^ can greatly improve the permeance of the cross-linking membrane. Considering potential influence of Mg^2+^ concentration on cross-linking, we prepared GO membranes with a series of concentrations (from 0.25 M to 0.025 M) of Mg^2+^, which were named as GO_HDA–0.25Mg^2+^_, GO_HDA–0.125Mg^2+^_, GO_HDA–0.05Mg^2+^_, and GO_HDA–0.025Mg^2+^_, respectively. As shown in Fig. 4a–c, we used GO_HDA–Mg^2+^_ membranes, which have a thickness of about 200 nm, to investigate the permeation with different concentrations of Mg^2+^. As the concentration of Mg^2+^ decreased from 0.25 M to 0.025 M, the water fluxes gradually reduced from 142.2 to 71.5 L m^−2^ h^−1^ bar^−1^ for methylene blue, from 114.4 to 70.3 L m^−2^ h^−1^ bar^−1^ for pararosaniline, and from 114.2 to 71.6 L m^−2^ h^−1^ bar^−1^ for rhodamine B, respectively, while the rejections remained constant. In the case of the lowest Mg^2+^ concentration of 0.025 M, the water fluxes were still about seven times that without Mg^2+^. It further indicated that Mg^2+^ plays a key role in improving the water flux of cross-linking GO membrane.

In addition, the effect of thickness of membrane on water flux and reject rate were also explored, as shown in Fig. 4d. The thickness of membrane can be controlled by the amount of GO suspension loaded on the substrate. GO_HDA–0.25Mg^2+^_ membranes with four thickness of 100 nm, 200 nm, 300 nm and 400 nm, were prepared by using 1.5 mL, 3 mL, 4.5 mL and 6 mL of mixture GO solution. With increasing of the thickness of the membrane, the water flux decreases dramatically from 251.5 to 90.2 L m^−2^ h^−1^ bar^−1^ while the rejection remained constant, suggesting that the highest water flux could be further improved without sacrificing dyes rejection by controlling membrane thickness.

### The cation–π interactions between hydrated Mg^2+^ and GO sheets

**Fig. 5:**
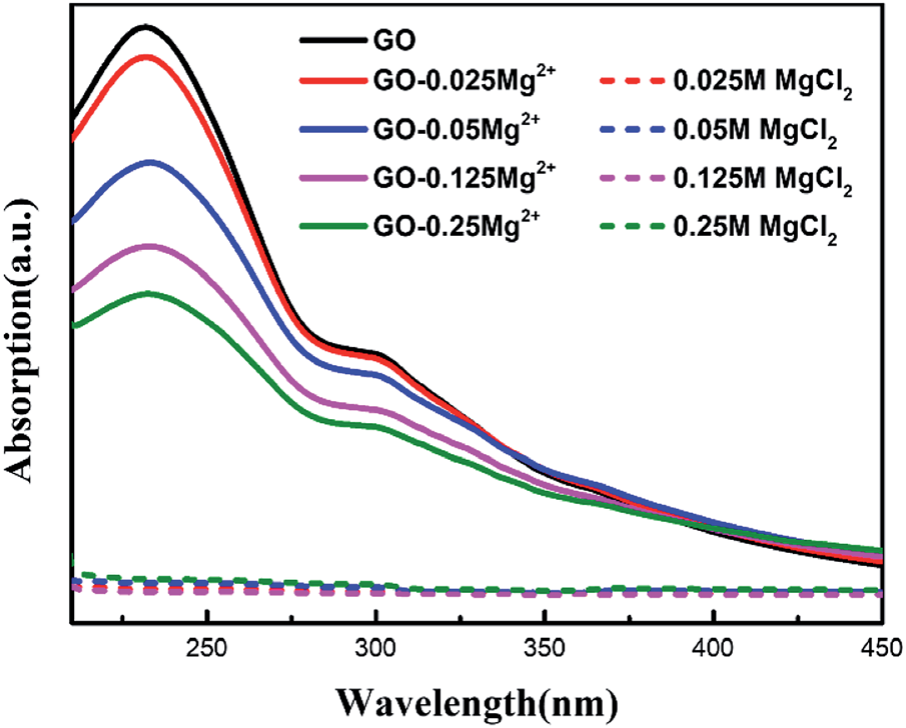
UV absorption spectra of GO suspension (100 mg L^−1^) 1 : 1 mixed with 0.025 M, 0.125 M, 0.05 M and 0.25 M Mg^2+^ solutions.

To illustrate the underlying physical mechanism of Mg^2+^ taking place in the process, we performed UV absorption spectra of GO solutions mixed with 0.25 M, 0.125 M, 0.05 M, and 0.025 M Mg^2+^, respectively. As shown in Fig. 5, the characteristic peak at ∼230 nm was attributed to the π–π* from the aromatic double bond conjugate.^13,16,31^ Compared with the GO in pure water, the intensity of GO mixed with Mg^2+^ solution was significantly decreased. It is showed that the trend was positively correlated with the concentration of Mg^2+^ solution. It indicated that the conjugated double bonds of aromatic groups in GO are greatly influenced by the concentration of salt solution.^11^ Our previous density functional theory computations show that high multivalence metal ions should have a strong cation–π interaction with the graphene sheet, resulting in ions enrichment on surface of graphene.^16^ Hence, the enrichment of Mg^2+^ on GO sheets based on the strong cation–π interaction, can promoted reasonable cross-linking and improved the water channels of membrane in terms of flatness and low mass transfer resistance. As a result, the GO_HDA–Mg^2+^_ membrane has ultrahigh water flux, while keeping high dyes rejection.

## Conclusions

In summary, we successfully enhanced the permeance of the cross-linking GO membrane by using the cation–π interaction between Mg^2+^ and aromatic ring structure. The enhancement is positively correlated with the concentration of Mg^2+^. It not only overcomes the swelling of GO membrane and enhances the stability of membrane, but also solves limitation of low water flux. This is attributed to the presence of Mg^2+^, which can prevent excessive chemical cross-linking and improves the water channels of membrane in terms of flatness and low mass transfer resistance surface during the cross-linking reaction. Therefore, the GO_HDA–0.25Mg^2+^_ exhibits ultrahigh water flux (143.2 L m^−2^ h^−1^ bar^−1^) and high reject rate for dyes in the filtration experiment. The GO_HDA–Mg^2+^_ membranes also have an outstanding stability in filtration process, which is attributed to the C–N bond formed between GO and HDA. This study suggests that other ions that have strong cation–π interaction, could have a similar effect like Mg^2+^, may opens a new door for the chemical cross-linking mode of GO membrane combined with ions.

## Conflicts of interest

There are no conflicts to declare.

## Supplementary Material

RA-009-C9RA07109A-s001
