# Supplementary material for: Ultrahigh permeance of a chemical cross-linked graphene oxide nanofiltration membrane enhanced by cation–π interaction
Source: RSC Adv. 2019 Dec 6;9(69):40397–403. doi: 10.1039/c9ra07109a (PMC9076233; doi:10.1039/c9ra07109a)
Supplement: RA-009-C9RA07109A-s001 [file RA-009-C9RA07109A-s001.pdf]

Electronic Supplementary Material (ESI) for RSC Advances.
This journal is © The Royal Society of Chemistry 2019

Ultrahigh permeance of chemical cross-linking graphene oxide nanofiltration membrane enhanced by cation-π interaction

Ruobing Yi,[a] Rujie Yang,[b] Risheng Yu,[b] Jian Lan,[c] Junlang Chen,[b] Zhikun Wang,[b] Liang Chen,[b,#] Minghong Wu[a,#]

[a.] Shanghai Applied Radiation Institute, Shanghai University, Shanghai 200444, P. R. China. E-mail: mhwu@shu.edu.cn (M.W.)

[b.] Department of Optical Engineering, Zhejiang A&F University, Lin'an, Zhejiang 311300, P. R. China. E-mail: liang_chen05@126.com (L.C.)

[c.] College of Pharmaceutical Chemistry and Materials Engineering, Taizhou University, Taizhou, Zhejiang 317000, China.

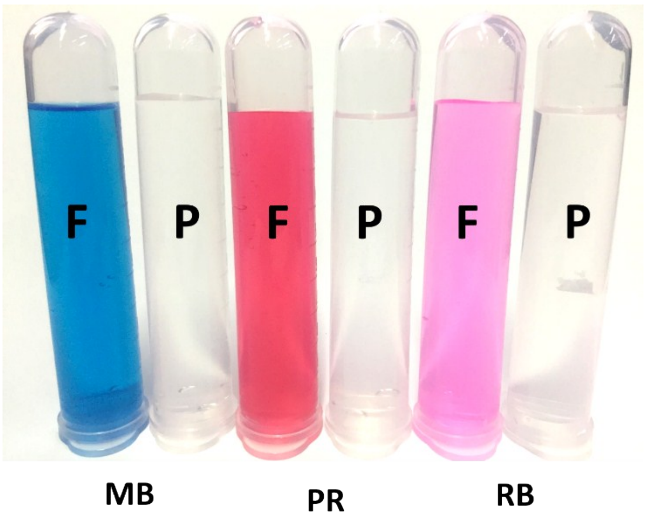

Fig. S1 Photographs of feed and permeate solutions containing different dye molecules before and after filtration. F and P represent feed and permeation solution, respectively.

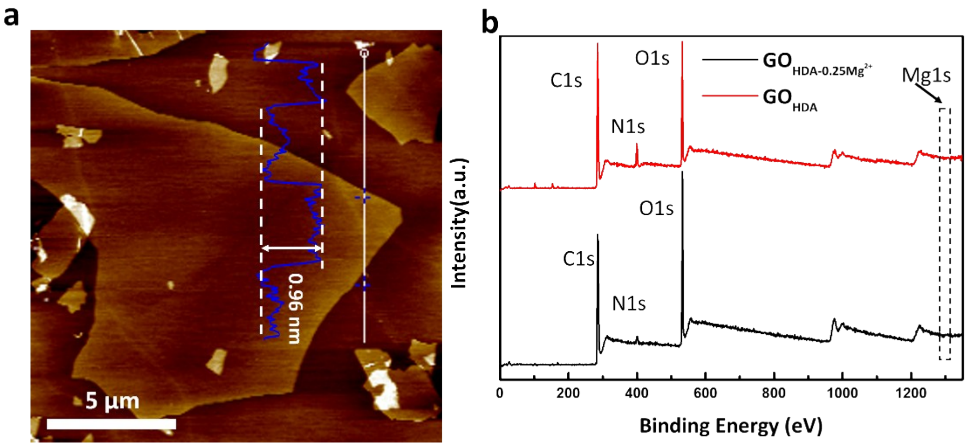

Fig. S2 (a) AFM image of GO flakes. (b) The XPS survey spectra of $GO_{HDA\text{-}0.25Mg^{2+}}$ and GO_{HDA}

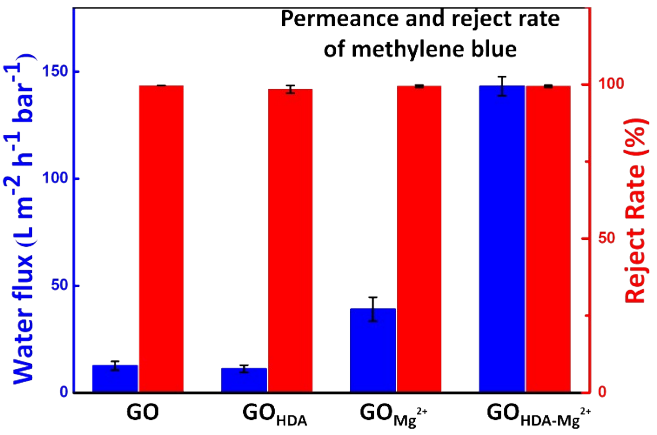

Fig. S3 Water flux and reject rate of GO, GO$_{HDA}$, GO$_{Mg^{2+}}$ and GO$_{HDA-Mg^{2+}}$ membranes for rejection of

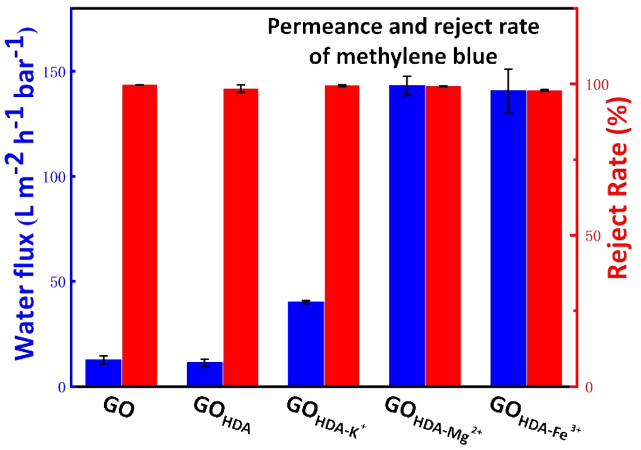

methylene blue at a pressure of 1.0 bar.

Fig. S4 Water flux and reject rate of GO, GO$_{HDA}$, GO$_{HDA-k^{+}}$, GO$_{HDA-Mg^{2+}}$ and GO$_{HDA-Fe^{3+}}$ membranes for rejection of methylene blue at a pressure of 1.0 bar.
